# Acute Aerobic Exercise at Different Intensities Modulates Motor Learning Performance and Cortical Excitability in Sedentary Individuals

**DOI:** 10.1523/ENEURO.0182-23.2023

**Published:** 2023-11-16

**Authors:** Hsiao-I Kuo, Ming-Hsien Hsieh, Yi-Ting Lin, Michael A. Nitsche

**Affiliations:** 1 School and Graduate Institute of Physical Therapy, National Taiwan University, Taipei 10055, Taiwan; 2 Department of Rehabilitation, National Taiwan University Hospital, Taipei 10055, Taiwan; 3 Department of Psychiatry, National Taiwan University Hospital, Taipei 10055, Taiwan; 4 Department Psychology and Neurosciences, Leibniz Research Center for Working Environment and Human Factors, 44139 Dortmund, Germany; 5 Bielefeld University, University Hospital OWL, Protestant Hospital of Bethel Foundation, University Clinic of Psychiatry and Psychotherapy and University Clinic of Child and Adolescent Psychiatry and Psychotherapy, 33615 Bielefeld, Germany

**Keywords:** aerobic exercise, cortical excitability, motor learning, sedentary

## Abstract

Converging evidence indicates the beneficial effects of aerobic exercise on motor learning performance. Underlying mechanisms might be an impact of aerobic exercise on neuroplasticity and cortical excitability. Evidence suggests that motor learning and cortical excitability alterations correlate with the intensity of aerobic exercise and the activity level of participants. Thus, this study aims to investigate the effects of different aerobic exercise intensities on motor learning and cortical excitability in sedentary individuals. The study was conducted in a crossover and double-blind design. Twenty-six healthy sedentary individuals (13 women and 13 men) performed a motor learning task and received a cortical excitability assessment before and after a single session of low-, moderate-, and high-intensity aerobic exercise or a control intervention. The study revealed that motor learning performance and cortical excitability were significantly enhanced in the moderate-intensity aerobic exercise, compared with the other conditions. These findings suggest aerobic exercise intensity-dependent effects on motor learning in sedentary adults. The underlying mechanism might be an exercised-induced alteration of cortical excitability, specifically a reduction of GABA activity.

## Significance Statement

Acute aerobic exercise enhances motor learning and cortical excitability in sedentary individuals. The study found that moderate-intensity aerobic exercise is the most favorable condition to reap the largest benefits. Furthermore, the obtained intervention-dependent learning improvement might be caused primarily by GABA reduction.

## Introduction

Converging evidence suggests that aerobic exercise positively influences brain functions and benefits mental health and neurologic diseases (Dimeo et al., 2001; Chang et al., 2012; Heijnen et al., 2015; Suarez-Manzano et al., 2018). Underlying mechanisms might include an impact of aerobic exercise on neuroplasticity and cortical excitability (Arcangelo et al., 2017; Cabral et al., 2019). Transcranial magnetic stimulation (TMS) techniques provide a noninvasive way to assess the impact of aerobic exercise on cortical excitability of the human motor cortex (Smith et al., 2014; Morris et al., 2020). Previous TMS studies have shown that acute aerobic exercise increases cortical facilitation and reduces cortical inhibition (Singh et al., 2014; Smith et al., 2014). Furthermore, studies have shown that this cortical excitability change is associated with improvements of motor learning (Kuo et al., 2023).

Although these findings support the clinical usefulness of aerobic exercise interventions, discrepant outcomes have also been documented. While a previous study found improvements of motor skill acquisition by aerobic exercise (Thomas et al., 2016), others have reported no benefit on motor learning (Singh et al., 2016; Snow et al., 2016). Although numerous factors might contribute to this heterogeneity, exercise intensity may play a key role. Some studies found that both high- and low-intensity aerobic exercise increased cortical excitability (Opie and Semmler, 2019), while another study reported that only moderate-intensity, but not low-intensity, cycling enhanced cortical excitability (MacDonald et al., 2019). Some studies, however, even describe no change of cortical excitability following low- and high-intensity aerobic exercise (Smith et al., 2014; Andrews et al., 2020). Since aerobic exercise intensity is a major confounder when comparing different studies, and the impact of interventions on plasticity, which is assumed to underlie respective learning effects, is nonlinear dosage dependent (Monte-Silva et al., 2010; Batsikadze et al., 2013; Mosayebi-Samani et al., 2020), systematic knowledge of these aspects is important.

Results of a recent study suggest that participants’ usual daily activity level (e.g., baseline activity) also affects the effects of acute aerobic exercise-induced cortical excitability alterations (Lulic et al., 2017). Many studies have used TMS to explore acute effects of aerobic exercise on cortical excitability; however, most studies did not document participants’ baseline physical activity levels (Opie and Semmler, 2019). The sedentary lifestyle became more and more frequent worldwide during and after the COVID pandemic (Celis-Morales et al., 2020). This lifestyle leads to deficits of cognitive and motor learning performance, and to mental and neurologic diseases (Thomée et al., 2012; Huang et al., 2022). Therefore, it is important to gain insight into how aerobic exercise improves performance in these conditions, and via which mechanisms. The purpose of this study is thus to explore motor learning and cortical excitability alterations following different controlled aerobic exercise intensities (to identify optimal conditions for cortical activation) in sedentary adults. Aerobic exercise involved 30 min of continuous low-, moderate-, or high-intensity cycling. Motor learning performance was assessed by the serial reaction time task (SRTT), a standard paradigm to test implicit motor sequence learning (Nissen and Bullemer, 1987; Wulf, 2013). Cortical excitability was monitored by TMS paradigms. We hypothesized that motor learning performance and cortical excitability are enhanced after aerobic exercise interventions (low-, moderate-, and high-intensity exercise) compared with no exercise. Additionally, we expected that aerobic exercise would show nonlinear and dosage-dependent associated effects on brain physiology and motor learning performance. Since our foregoing study found that exercise-dependent physiological alterations are associated with motor learning improvement (Kuo et al., 2023), we expected a similar association between exercise-induced cortical excitability alterations and improvement of motor learning also in the present study.

## Materials and Methods

### Participants

The study recruited 26 (13 women and 13 men) healthy right-handed (screened by Edinburgh Handedness Scale), and sedentary participants (determined by taking part in <150 min physical activity per week for the 6 months period preceding study participation (Hendy et al., 2022). The mean (SD) age of the participants was 27.2 (1.01) years. No participant had a history of psychiatric or neurologic diseases; was pregnant or had metallic head implants; received any medication, physical, and cognitive training program; or consumed nicotine during the study period. The physical activity level was assessed by the International Physical Activity Questionnaire. This study was approved by the Research Ethics Committee of National Taiwan University Hospital (approval #202105119RIND) and conforms to the Declaration of Helsinki. Written informed consent was obtained from all participants before inclusion in the study. The demographic characteristics of the participants are summarized in Table 1.

**Table 1:** Demographic characteristics of participants

Group size	*n* = 26
Gender (male/female)	13/13
Age	27.2 (1.01)
BMI	24.3 (3.2)
PA total score	3222 (1361)

PA, Physical activity level assessed by the International Physical Activity Questionnaire, expressed in metabolic equivalent of task (MET)-minutes/wk. All data are presented as the mean (SD).

### Exercise intervention

The participants were asked to pedal a stationary exercise bike. At the beginning of each intervention session, participants were seated in a comfortable chair and allowed to rest for 5 min before measuring baseline heart rate (HR; measured for 1 min) via a heart rate strap (model H7, Polar). Exercise intensity was determined on the basis of the individual age-predicted maximum HR (HRmax; i.e., 220 − age). For low-, moderate-, and high-intensity exercise, HR was controlled between 40% and 60%, between 61% and 74%, and between 75% and 95% of the HRmax, respectively. Exercise intensity ranges were chosen in accordance with the findings of previous studies exploring the association of exercise with cognitive performance in healthy adults (Baltar et al., 2018; Herold et al., 2019). The exercise included warm-up for 5 min, main exercise at the target HR for 20 min and cool-down for 5 min. Throughout the exercise period, HR was monitored continuously. The protocol was considered to be effective and suitable in a previous study (Herold et al., 2019). Under the control condition, participants were asked to rest for 30 min. During this period, participants could interact with the study staff, use their smartphones, or read, but were not allowed to conduct whole-body movements. HR was assessed also during the rest condition.

### Serial reaction time task: motor learning

**Figure 1.**
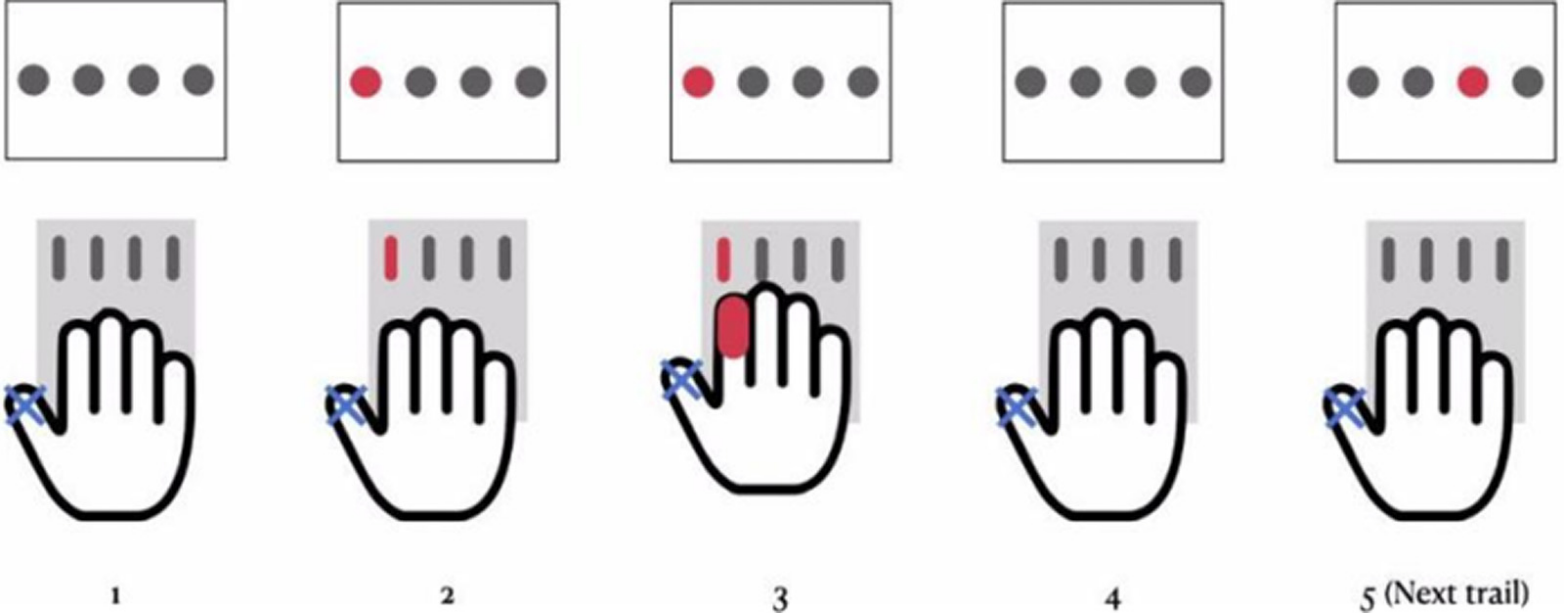
Schematic drawing of the SRTT. (1) A signal appeared at one of the four positions, which were horizontally spaced on a computer screen. (2) The participants were required to press each button of the response pad with a different finger of the right hand (the index finger for button 1, the middle finger for button 2, the ring finger for button 3, and the little finger for button 4). (3) The participants were asked to push the key corresponding to the position of the signal as quickly and correctly as possible. (4) After a button was pushed, the signal disappeared. (5) The SRTT progressed to the next stimulus.

The SRTT is a well introduced instrument to explore motor sequence learning in humans (Robertson, 2007). Participants were asked to sit in front of a computer screen at eye level, and a response pad with four buttons (numbered from 1 to 4) was placed on a desk. They were required to press each button of the response pad with a different finger of the right hand (the index finger for button 1, the middle finger for button 2, the ring finger for button 3, and the little finger for button 4). In each trial, a signal (asterisk) appeared at one of four positions that were horizontally spaced on a computer screen and permanently marked by horizontal lines. The participants were asked to push the key corresponding to the position of the signal (asterisk) placed above these lines as quickly and correctly as possible. After the button was pushed, the signal (asterisk) disappeared. The SRTT progressed to the next stimulus independent of the correctness of the pressed key (Fig. 1). Participants were not informed regarding the correctness of their reactions. A test session included eight blocks of 120 trials each. In blocks 1 and 6, the sequence of signals (Fig. 1, asterisks) followed a pseudorandom order, and signals (Fig. 1, asterisks) were presented equally frequently in each position and never in the same position in two subsequent trials. In the remaining blocks (blocks 2–5, 7, and 8), an identical sequence of signal (Fig. 1, asterisk) positions, which consisted of 12 trials, was presented and repeated 10 times in each of the blocks; thus, participants learned this repeated sequence. The outcome measures were response time (RT) and error rate (ER). RT was recorded from the appearance of the go signal until the first button was pressed. An individual ER was calculated by the sum of incorrect responses for each block for each participant in each session. Differences in outcome measures between blocks 5 and 6 represent a pure measure of sequence-specific learning, because in these blocks, sequence-independent task learning has been completed (Nitsche et al., 2003), and thus any performance differences between these blocks can be attributed to the sequence. Divergent versions of tasks with different sequences per session and pre-post aerobic exercise were conducted, and the task versions were also randomized to intervention conditions before and after exercise.

### TMS: motor cortical excitability

TMS over the left primary motor cortex was applied to induce motor evoked potentials (MEPs) of the right abductor digiti minimi muscle (ADM). The ADM is a well introduced model muscle, which allows the monitoring of MEPs originating selectively from this muscle, and, furthermore, it is relatively unproblematic for participants to completely relax this muscle (Barsi et al., 2008). Single-pulse TMS was applied by a magnetic stimulator (model 200, Magstim) with a figure-of-eight magnetic coil (diameter of one winding, 70 mm; peak magnetic field, 2.2 T). The TMS coil was held tangentially to the skull, with the handle pointing backward and laterally at 45° from midline. The hot spot (individual optimal coil position) was defined as the site where TMS with medium intensity resulted in the largest stable MEP of the target muscle and marked with a water-resistant felt marker. The hot spot was consistently maintained preintervention and postintervention, and across intervention days. Surface electromyography was recorded from the right ADM via Ag-AgCl electrodes in a belly tendon montage. The signals were amplified, and bandpass filtered (2 Hz to 2 kHz; sampling rate, 5 kHz). Resting motor threshold (RMT) was determined as the minimum TMS intensity that elicited a peak-to-peak MEP of 50 μV in the relaxed muscle in at least 5 of 10 consecutive trials. Active motor threshold (AMT) was the minimum intensity eliciting an MEP response of ∼200–300 μV during spontaneous moderate background muscle activity (∼15% of maximum muscle strength) in at least 5 of 10 consecutive trials. Motor thresholds reflect neuronal membrane excitability and depend primarily on ion channels, since they are increased by voltage-gated sodium channel blockers, but are not modulated by substances affecting GABAergic or glutamatergic transmission (Ziemann et al., 1996a, 1998). Input-output (I-O) curves were obtained by four different stimulus intensities (100, 110, 130, and 150% of RMT), each with 15 pulses. With increased stimulus intensities, amplitudes of motor evoked potentials also increase, thus reflecting recruitment of a larger neuronal pool, including recruitment of neurons farther away from the stimulating coil (Chen, 2000; Grundey et al., 2013; Kuo et al., 2016). Intracortical excitability was monitored by short-interval intracortical inhibition (SICI) and intracortical facilitation (ICF). For obtaining SICI/ICF, a paired-pulse TMS protocol was used. Paired-pulse TMS was applied by connecting two stimulators (model 200, Magstim) via a BiStim module. The first subthreshold conditioning stimulus (CS) was set to 80% AMT (Grundey et al., 2013). The second stimulus [test stimulus (TS)] was set to elicit an MEP size of ∼1 mV (Grundey et al., 2013). Regarding the intensity of the conditioning stimulus, we applied 80% of AMT, which follows previous studies that explored the effects of aerobic exercise on cortical excitability (Smith et al., 2014; Lulic et al., 2017; Morris et al., 2020). TMS with this conditioning stimulus intensity (SI) did not elicit MEPs in the present study. We applied interstimulus intervals (ISIs) of 2, 3, 5, 10, and 15 ms to determine SICI and ICF (Grundey et al., 2013). The pairs of stimuli were combined in 15 blocks, where each ISI was represented once, together with one additional single test pulse in pseudorandomized order. The first three ISIs (2, 3, 5 ms) induce inhibitory effects, and the last two ISIs (10, 15 ms) induce facilitatory effects, which are controlled by inhibitory and excitatory interneurons (Kujirai et al., 1993; Kuo et al., 2023). SICI is primarily controlled by the GABAergic system, but to a smaller amount reflects also activity of the glutamatergic system (Ilić et al., 2002). ICF is primarily determined by the glutamatergic system, but to a smaller amount also involves GABA (Ziemann et al., 1996b).

### Experimental procedure

**Figure 2.**
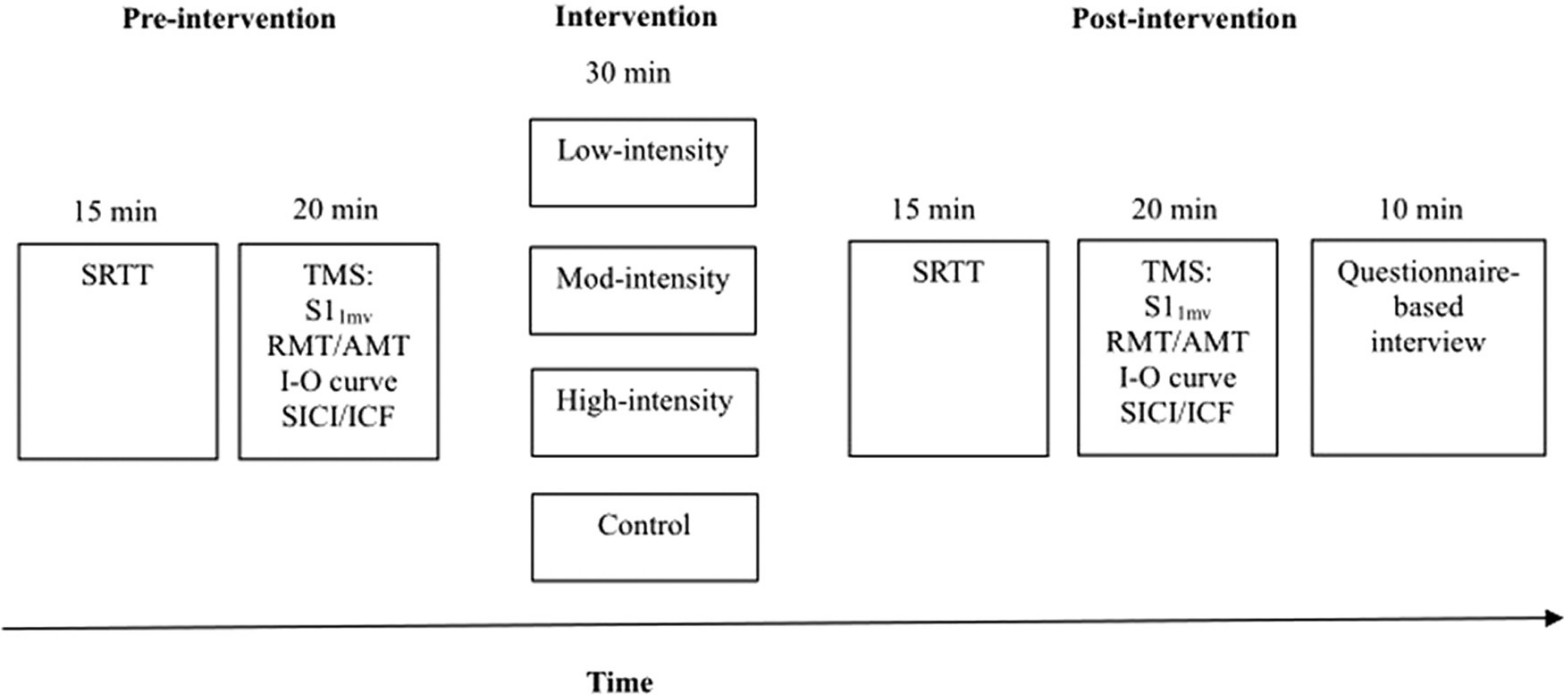
Experimental course of the study. Participants took part in four experimental sessions. SRTT (motor learning performance) and TMS measurements (corticospinal, and cortical excitability) were assessed before and after interventions (low-intensity, moderate-intensity, high-intensity exercise, and control condition). A questionnaire-based interview (asking whether the participants felt fatigue, headache, or dizziness) was conducted after the final assessments for each exercise intervention.

Each participant took part in four experimental sessions in a random order. Each session was separated by at least 1 week to avoid carryover effects. Each session consisted of the following procedures (Fig. 2): 15 min motor learning assessment (SRTT); 20 min cortical excitability measurements (TMS protocols); and a 30 min aerobic exercise intervention (low-, moderate-, and high-intensity) or a control intervention followed by a repetition of SRTT and TMS assessments. A 10 min questionnaire-based interview (asking whether the participants felt fatigue, headache, or dizziness) was conducted after final assessments for each exercise intervention. For cortical excitability assessments, participants were seated in a comfortable chair with head and arm rests. The hotspot of the right ADM was determined over the left primary motor cortex, and 20 MEPs were recorded with the TMS intensity, which elicited on average an MEP of 1 mV amplitude (SI_1mV_; the stimulus intensity required to evoke a MEP with a mean peak-to-peak amplitude of ∼1mV)). After measuring SI_1mV_, RMT and AMT were obtained with standard procedures. Afterward, a 15 min break followed to prevent a possible effect of muscle contraction on the next assessment. After this break, the I-O curve and SICI-ICF were determined in randomized order. These TMS parameters were conducted before and after intervention.

### Data analysis

Aerobic exercise characteristics (HR and percentage of HRmax) were analyzed via a repeated-measures ANOVA with the within-subject factors intervention condition (low-, moderate-, high-intensity aerobic exercise and control).

With regard to SRTT, for each block of trials, mean RT was calculated for each individual separately. Incorrect responses, RTs of <200 or >3000 ms, and those that were above 3 SDs different from the individual mean RT were excluded. The mean RT was standardized to that of block 1 for each participant in each intervention condition separately to control for initial RT differences. Additionally, the SD of RTs for each subject in every block was calculated as an index of RT variability. Statistical analyses were performed for the outcome measures of the cognitive task (RT, standardized RT, variability, and ER) via an ANOVA (level of significance, 0.05 for all statistical tests), with the within-subject factors intervention condition (low-, moderate-, high-intensity aerobic exercise and control), time (preintervention and postintervention), and block (for SRTT). To explore a priori differences between conditions, secondary ANOVAs were conducted to compare the baseline outcome measurements between intervention conditions (low-, moderate-, high-intensity aerobic exercise and control). Because for SRTT, RT differences between block 5 and 6 show an exclusive measure of sequence motor learning (Pascual-Leone et al., 1994), as task routine is thought to be equivalent in both blocks, hence any differences in performance should be because of implicit sequence learning, Fisher’s least significant difference (LSD) tests were applied to compare differences of respective RTs between these blocks. Moreover, we conducted an analysis of covariance (ANCOVA) for the exercise intervention condition with order as a covariate to rule out systematic effects of the order of intervention conditions on the results.

For SI_1mV_, RMT, and AMT, the individual means of the TMS intensity at SI_1mV_, RMT, and AMT were calculated for the intervention conditions, respectively. Repeated-measures ANOVAs were conducted for the above-mentioned data with RMT, AMT, and SI_1mV_ values as the dependent variables, and intervention condition (low-, moderate-, high-intensity aerobic exercise and control), and time (preintervention and postintervention) as within-subject factors. With regard to the I-O curve, the individual means of MEP amplitudes resulting from respective TMS intensities, and exercise condition combinations were analyzed for all subjects. Repeated-measures ANOVAs were performed for the above-mentioned data using MEP amplitudes as the dependent variable, and intervention condition (low-, moderate-, high-intensity aerobic exercise and control), time (preintervention and postintervention), and TMS intensity as within-subject factors. For SICI-ICF, the percentage change of CS MEP from unconditioned (TS) cortical reactivity (TS – CS/CS * 100) for each interstimulus interval were calculated. Furthermore, grand means of ISIs at 2, 3, and 5 ms for SICI and means of ISIs at 10 and 15 ms for ICF were analyzed. Repeated-measures ANOVAs were performed [with ISIs, intervention conditions, and time as within-subject factors, and percentage change of CS MEP from unconditioned (TS) cortical reactivity as the dependent variable] to explore exercise-dependent alterations. Similar to motor learning performance, secondary ANOVAs were conducted to compare the baseline MEP amplitudes between intervention conditions (low-, moderate-, high-intensity aerobic exercise and control) to explore any differences before interventions. The data obtained from SRTT and TMS passed the Shapiro–Wilk test (normal distribution) and Levene’s test (homogeneity of variance); thus, the statistical preconditions for conduction of an ANOVA were fulfilled. In case of significant results of the ANOVAs, Fisher’s LSD tests were applied to receive more detailed knowledge about the origin of the effects. All data are expressed as the mean (SD).

Simple bivariate correlations (Pearson’s *r* coefficient) were conducted to explore the relation between motor learning (absolute RT differences of blocks 5 and 6) and neurophysiological parameters (SI_1mV_, RMT, AMT, I-O curve: means of different intensities; SICI/ICF: means of different ISIs) in each intervention separately. All statistical analyses were performed using SPSS Statistics 9 (version 22; IBM).

## Results

In the questionnaire-based interview, two participants reported fatigue after the high-intensity exercise intervention; however, most participants tolerated the low-, moderate-, and high-intensity aerobic exercise protocols well. As shown in Table 2, there was a significant effect on intervention condition for HR (*F*_(3)_ = 4.23, *p* = 0.03) and percentage of HRmax (*F*_(3)_ = 3.13, *p* = 0.04).

**Table 2:** Comparison of aerobic exercise characteristics for different intervention conditions

	High intensity	Moderate intensity	Low intensity	Control
HR, bpm	162 (2)	125 (4)	96.9 (2)	82.1 (1)
HRmax, %	81.1 (1.7)	70.2 (3.5)	50.2 (1.2)	45.2 (2.4)

Data are presented as the mean (SD).

### SRTT

**Figure 3.**
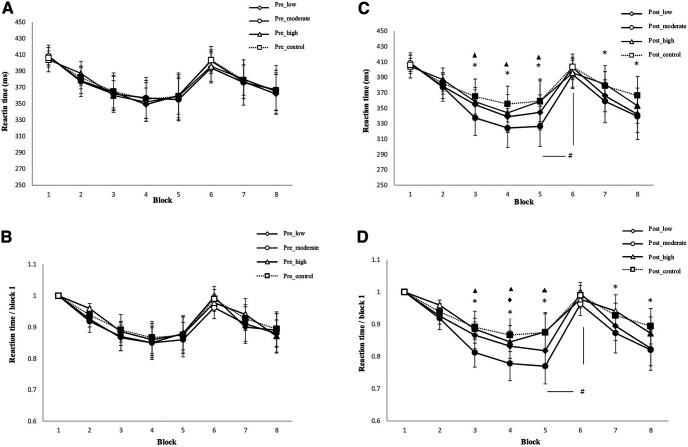
SRTT performance (reaction time) before and after the different intervention conditions: low-, moderate-, and high-intensity exercise or control. ***A***, ***B***, Depicted are the mean absolute reaction time (in ms) before intervention (***A***) and standardized reaction time before each intervention condition (blocks 1–8; ***B***). In blocks 1 and 6, random stimuli, and in the remaining blocks, the sequence were presented. ***C***, ***D***, Mean absolute reaction time (in ms) after intervention (***C***) and standardized reaction time after intervention (***D***). The results indicate that participants learned in all conditions (low-, moderate-, and high-intensity exercise, and control). In addition, reaction time was significantly shorter under the moderate-intensity exercise condition than under the low-intensity exercise condition (block 5), high-intensity exercise condition (blocks 3, 4, and 5), and control condition (blocks 3, 4, 5, 7, and 8) after, but not before intervention. For ***C*** and ***D***, the reaction time difference between blocks 5 and 6, which is a pure index of sequence motor learning, was larger for the moderate-intensity exercise condition compared with the control condition, indicating improved learning under moderate-intensity exercise after intervention. Filled symbols indicate significant reaction time differences within respective intervention conditions relative to block 1. Asterisks indicate significant differences between post_moderate-intensity exercise and the post_control condition for a single block. The floating triangle and diamond symbols indicate significant differences between post_moderate-intensity exercise and post_high-intensity/post_low-intensity exercise conditions within a block. The hash sign indicates a significant RT difference between blocks 5 and 6 with respect to the post_moderate-intensity exercise versus the post_control condition. Error bars in this and the following figures represent the SEM.

As shown in Tables 3 and 4, for absolute RT, the repeated-measures ANOVA showed significant main effects of the factors block (*F*_(7)_ = 8.323, *p* = 0.006), condition (*F*_(3)_ = 9.613, *p* = 0.004), and time (*F*_(1)_ = 5.31, *p* = 0.01), and significant condition × block (*F*_(21)_ = 51.49, *p* < 0.001), block × time (*F*_(7)_ = 10.24, *p* = 0.002), condition × time (*F*_(3)_ = 5.53, *p* = 0.01), and block × condition × time (*F*_(7)_ = 9.1, *p* = 0.005) interactions. For standardized RT, the results revealed significant main effects of the factors block (*F*_(7)_ = 501.55, *p* < 0.001), condition (*F*_(3)_ = 502.12, *p* < 0.001), and time (*F*_(1)_ = 50.2, *p* < 0.001), and significant condition × block (*F*_(21)_ = 531.24, *p* < 0.001), block × time (*F*_(7)_ = 44.21, *p* < 0.001), condition × time (*F*_(3)_ = 50.62, *p* < 0.001), and block × condition × time (*F*_(21)_ = 52.76, *p* < 0.001) interactions. For absolute RT, the main effect of block was caused by reduced RTs in all conditions compared with random block 1 in later sequences, with the exception of the random block 6, which did not contain the learned sequence (Fig. 3). The main effects of condition and time resulted from significantly shorter RTs observed under the post_moderate-intensity aerobic exercise condition than under the post_low-intensity and post_high-intensity aerobic exercise condition, and the post_control condition (Fig. 3*C*). The results with standardized RT were comparable. Standardized RT were significantly shorter under the post_moderate-intensity aerobic exercise condition than under the post_low-intensity and post_high-intensity aerobic exercise conditions, and post_control condition (Fig. 3*D*). Regarding significant interactions, the *post hoc t* tests indicated that the RT difference between block 6 (RT6) and RT5 was significantly larger under the post_moderate-intensity aerobic exercise condition than the pre_moderate-intensity aerobic exercise conditions (absolute RT, *p* < 0.001; standardized RT, *p* = 0.003), and the post_control condition (absolute RT, *p* = 0.001; standardized RT, <0.001), implying improved motor learning after moderate-intensity aerobic exercise intervention. Significant larger RT6–RT5 differences were moreover observed under the post_moderate-intensity condition versus the post_low-, and post_high intensity aerobic exercise condition (absolute RT, *p* = 0.001; standardized RT, *p* = 0.003)/post_high-intensity aerobic exercise condition (absolute RT, *p* = 0.001; standardized RT, *p* = 0.003). However, no significant RT6–RT5 differences were observed under the post_low/post_high-intensity aerobic exercise conditions versus the post_control condition (*p* > 0.05). With regard to the secondary ANOVAs conducted for RTs before intervention, the results showed a significant main effect of block for both RT (*F*_(7)_ = 8.21, *p* < 0.001) and standardized RT (*F*_(7)_ = 4.21, *p* = 0.02). For ER and variability, the repeated-measures ANOVAs revealed no significant main effects of condition, block, or their interactions (all *p* > 0.05; Tables 3, 4). Furthermore, the result of the ANCOVA did not reveal a significant effect of order (*p* = 0.335).

**Table 3:** Repeated-measures ANOVA results for motor learning (SRTT)

Parameters	Conditions	df	*F* value	*p* value
SRTT				
RT (absolute)	Block	7	8.323	**0.006**
	Condition	3	9.613	**0.004**
	Time	1	5.31	**0.01**
	Condition × block	21	51.49	**<0.001**
	Block × time	7	10.24	**0.002**
	Condition × time	3	5.53	**0.01**
	Block × condition × time	7	9.1	**0.005**
RT (standardized)	Block	7	501.55	**<0.001**
	Condition	3	502.12	**<0.001**
	Time	1	50.2	**<0.001**
	Condition × block	21	531.24	**<0.001**
	Block × time	7	44.21	**<0.001**
	Condition × time	3	50.62	**<0.001**
	Block × condition × time	21	52.76	**<0.001**
Variability of RT	Block	7	1.24	0.32
	Condition	3	0.823	0.43
	Time	1	0.54	0.82
	Condition × block	21	0.89	0.48
	Block × time	7	1.62	0.21
	Condition × time	3	1.23	0.33
	Block × condition × time	21	0.9	0.41
Errors	Block	7	0.22	0.899
	Condition	3	0.913	0.58
	Time	1	1.32	0.26
	Intensity × block	21	0.95	0.52
	Block × time	7	2.1	0.09
	Condition × time	3	0.89	0.7
	Block × condition × time	21	0.82	0.72

The bold font indicates significant results (*p* < 0.05).

**Table 4:** Mean and SD of SRTT performance (reaction time, variability, and error rate) before and after the different intervention conditions (low-, moderate-, and high-intensity exercise or control)

SRTT	Low intensity				Moderate intensity				High intensity				Control			
	Before	After	Change	*p*	Before	After	Change	*p*	Before	After	Change	*p*	Before	After	Change	*p*
Reaction time																
Block 1	409.1 (13.2)	408.4 (13.4)	−0.7 (0.2)	0.174	407.1 (11.2)	408.2 (10.6)	1 (0.4)	0.267	408.2 (15.2)	404. (14.8)	−4.2 (1.2)	0.126	410.2 (21.3)	406.2 (8.5)	−4 (1.2)	0.264
Block 2	376.2 (21.3)	378.2 (15.7)	2 (1.3)	0.235	380.2 (20.2)	377.2 (18.4)	−3 (1.2)	0.176	378 (16.1)	387.1 (15)	9.1 (3.1)	0.33	375.2 (21.1)	382.1 (13.9)	6.9 (3.3)	0.124
Block 3	356 (13.5)	354.5 (19.2)	−1.5 (1.1)	0.176	358 (21.3)	337.2 (22.6)	−20.8 (3.1)	**<0.01**	355 (19.1)	358.3 (18.3)	3.3 (1.2)	0.34	360 (24.1)	365 (22.7)	5 (2.1)	0.233
Block 4	340 (21.2)	338.7 (20.3)	−1.3 (1.2)	0.418	339 (26.1)	324.2 (25.2)	−14.8 (2.2)	**0.001**	341 (21.2)	343.9 (23.7)	2.9 (2.2)	0.16	337 (23.1)	338 (23.2)	1 (2.2)	0.312
Block 5	341.2 (23.8)	344.3 (22.8)	3.1 (2.1)	0.328	327.2 (27.1)	312.6 (26.1)	−14.6 (3.1)	**0.001**	341 (30.1)	332.9 (28.8)	−8.1 (3.1)	**0.02**	341 (21.2)	359.1 (26.4)	18.1 (3.0)	0.234
Block 6	433.2 (21.2)	402.6 (13.6)	−21 (10.2)	0.242	435 (20.2)	393.8 (18.4)	−41.2 (11.2)	**<0.01**	431 (18.5)	395.8 (18.5)	−17.8 (1.9)	**0.01**	421 (20.2)	423 (16.6)	2 (1.8)	0.176
Block 7	321.3 (15.2)	318.1 (20.8)	−3.2 (2.5)	0.123	330.1 (28.2)	314 (27.8)	−16.1 (12.2)	**0.02**	323 (24.1)	328.2 (23.9)	5.2 (1.1)	0.21	321.3 (19.2)	319.2 (18.5)	−2.1 (1.8)	0.355
Block 8	322.1 (14.1)	319 (23.8)	−3.1 (1.9)	0.32	322 (31.1)	302 (30.0)	−20 (21.2)	**<0.01**	324 (23.1)	321.1 (22.9)	−2.9 (0.9)	0.16	322.1 (26.3)	321.3 (24.8)	−0.8 (0.2)	0.23
Variability																
Block 1	97.4 (48.2)	104.8 (25.6)	7.4 (3.2)	0.38	97.8 (32.2)	99.9 (51.2)	2.1 (1.2)	0.25	101 (22.2)	98.8 (31.5)	−2.2 (2.1)	0.09	101 (22.3)	104.8 (25.6)	3.8 (2.1)	0.07
Block 2	99.3 (32.0)	95.7 (60.2)	3.6 (1.8)	0.24	93.6 (30.1)	97.8 (22.3)	2.1 (1.1)	4.2	94.2 (30.2)	95.8 (33.2)	1.6 (0.2)	0.12	98.4 (33.8)	95.7 (60.2)	−2.7 (1.1)	0.77
Block 3	98.4 (36.8)	96.7 (45.1)	1.7 (1.1)	0.12	94.7 (31.3)	95.5 (37.2)	−1.2 (2.4)	0.8	97.7 (25.1)	93.2 (41.2)	−4.5 (1.1)	0.32	94.8 (47.1)	96.7 (45.1)	1.9 (0.9)	0.78
Block 4	94.9 (38.2)	99.6 (32.1)	4.7 (2.1)	0.32	99.2 (36.1)	98.6 (37.2)	−0.6 (0.8)	0.123	100.6 (42.1)	99.2 (36.1)	−1.4 (0.2)	0.26	99.9 (27.2)	99.6 (32.1)	−0.3 (1.1)	0.81
Block 5	96.6 (36.2)	98.9 (44.2)	2.3 (1.3)	0.27	98.8 (44.2)	95.1 (38.2)	−3.7 (1.1)	0.32	97.9 (42.2)	98.9 (31.3)	-0.1 (0.1)	0.31	96.1 (22.2)	98.9 (44.2)	2.8 (0.9)	0.51
Block 6	108.9 (44.3)	103.8 (80.9)	−5.1 (2.2)	0.12	105.8 (34.3)	105.3 (47.1)	−0.5 (2.3)	0.26	101.8 (79.9)	106.1 (24.2)	2 (1.1)	0.31	105.3 (47.1)	103.8 (80.9)	−1.5 (0.2)	0.21
Block 7	108.5 (44.2)	104 (67.3)	−4.5 (2.1)	0.23	103.9 (67.2)	104.2 (45.2)	−0.3 (0.3)	0.31	102 (54.1)	101.8 (53.3)	−4.3 (1.2)	0.54	104.2 (45.2)	104 (67.3)	−0.2 (0.1)	0.22
Block 8	107 (36.2)	108.9 (32.1)	1.9 (0.8)	0.61	104.2 (43.2)	101.2 (36.7)	−3 (2.1)	0.26	103.9 (22.1)	105 (33.1)	1.1 (2.2)	0.22	101.2 (36.7)	103.9 (32.1)	2.7 (1.1)	0.18
Error rate																
Block 1	4.04 (2.07)	4.1 (3.5)	0.06 (1.3)	0.70	4.1 (2)	4.2 (3.5)	0.1 (1.2)	0.8	4.05 (2.07)	4.2 (3.2)	0.15 (0.6)	0.56	4 (2.07)	4.1 (3.2)	0.1 (0.3)	0.31
Block 2	4.8 (2.2)	4 (2.9)	−0.8 (0.2)	0.77	4.4 (2.2)	4 (2.8)	−0.4 (0.4)	0.61	4.8 (2.2)	4.1 (3.1)	−0.7 (0.9)	0.21	4.3 (2.2)	4 (2.9)	−0.3 (1.1)	0.54
Block 3	4.4 (2.3)	4.3 (2.4)	−0.1 (0.1)	0.5	4.4 (1.3)	4.3 (2.4)	−0.1 (1.1)	0.25	4.4 (2.3)	4.2 (2.4)	−0.2 (0.1)	0.34	4.4 (2.3)	4.3 (2.3)	−0.1 (0.6)	0.22
Block 4	4.2 (1.1)	4.1 (2.4)	−0.1 (1.2)	0.56	4.3 (1.1)	4.2 (3.4)	−0.1 (0.4)	0.25	4.2 (1.1)	3.9 (2.2)	−0.3 (0.4)	0.67	4.1 (1.4)	3.9 (2.4)	−0.2 (1.2)	0.31
Block 5	4.8 (3.1)	3.9 (2.9)	−0.9 (0.9)	0.31	4.8 (3.1)	4.1 (2.2)	−0.7 (1.2)	0.31	5.1 (2.1)	4.1 (2.9)	−1 (0.1)	0.7	4.8 (2.9)	3.9 (2.9)	−0.9 (0.3)	0.31
Block 6	5.4 (3.4)	4.7 (3.1)	−0.7 (0.2)	0.51	5.8 (2.4)	4.8 (2.8)	−1 (0.9)	0.54	5.4 (3.4)	4.7 (2.8)	−0.7 (0.5)	0.77	5.4 (3.4)	4.7 (2.1)	−0.7 (0.6)	0.54
Block 7	4.9 (3.9)	4 (2.8)	−0.9 (0.3)	0.21	5 (2.9)	4.2 (2.7)	−0.8 (1.3)	0.22	5.1 (3.9)	4.8 (2.8)	−0.3 (0.2)	0.78	4.9 (3.9)	4.2 (2.9)	−0.7 (0.2)	0.22
Block 8	4.7 (1.7)	3.9 (2.1)	−0.8 (1.2)	0.22	4.7 (1.7)	4 (2.7)	−0.7 (0.4)	0.21	4.2 (2.7)	4 (2.1)	−0.2 (1.2)	0.81	4.7 (1.7)	4.1 (2.1)	−0.6 (0.5)	0.23

Significant results as pre-post comparison at *p* < 0.05 are shown in bold.

### Cortical excitability

**Figure 4.**
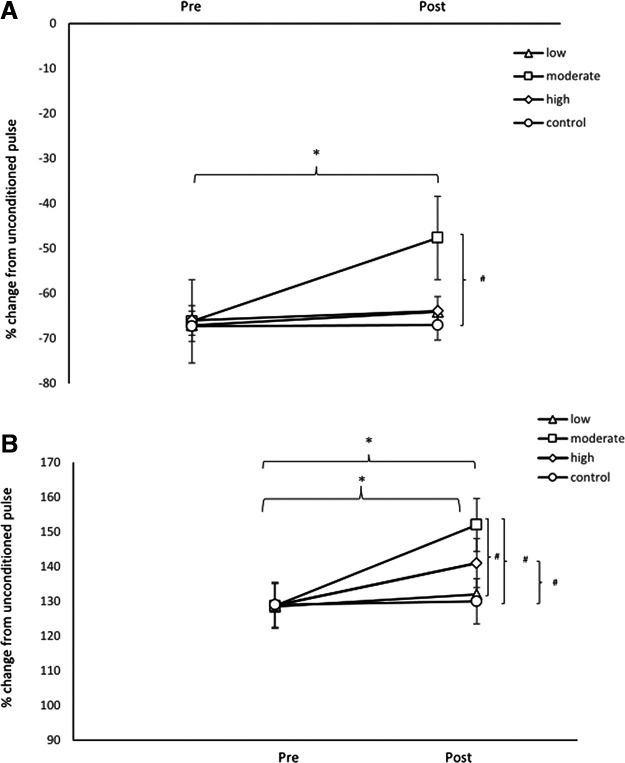
***A***, The preintervention to postintervention changes of the group means (the average SICI over all interstimulus intervals) are shown. Before intervention, the groups did not differ from each other. In the post_moderate-intensity aerobic exercise condition, inhibition was significantly decreased, compared with the pre_moderate-intensity aerobic exercise and post_control conditions. ***B***, The preintervention to postintervention changes of the group means (the average ICF over all interstimulus intervals) are shown. Before intervention, the groups did not differ from each other. In the post_moderate-intensity exercise condition, ICF was significantly enhanced versus the pre_moderate-intensity intervention, post_low-intensity exercise, and post_control conditions. In the post_high-intensity aerobic exercise condition, facilitation was significantly increased compared with the pre_high-intensity condition and post_control condition. The asterisks indicate significant differences between pre_intervention and post_intervention conditions. The hash symbols indicate significant differences between respective postconditions (*p *<* *0.05). See Extended Data Figure 4-1 for more details.

SI_1mV_, motor thresholds (AMT and RMT), and the I-O curve showed no significant effects of the main factor intervention condition and time or the respective interaction (*p* > 0.05; Tables 5, 6). With respect to SICI and ICF, the ANOVA revealed significant main effects of condition (*F*_(1)_ = 8.56, *p* < 0.001), ISI (*F*_(4)_ = 3.18, *p* = 0.001), time (*F*_(1)_ = 7.32, *p* < 0.001), and the condition × ISI (*F*_(4)_ = 7.21, *p* < 0.001), condition × time (*F*_(1)_ = 9.3, *p* <0.001), ISI × time (*F*_(4)_ = 8.3, *p* < 0.001), and condition × ISI × time (*F*_(4)_ = 7.78, *p* < 0.001) interactions were significant (Table 5). Before intervention, the baseline TMS data and secondary ANOVAs showed no significant differences between intervention conditions (all *p* > 0.05). After intervention, in the post_moderate-intensity aerobic exercise condition, inhibition was significantly decreased, compared with the pre_moderate-intensity aerobic exercise and postcontrol conditions (Fig. 4, Extended Data Fig. 4-1). Furthermore, in the post_moderate-intensity exercise condition, ICF was significantly enhanced versus the pre_moderate-intensity intervention, post_low intensity exercise, and post_control conditions. In the post_high-intensity aerobic exercise condition, facilitation was significantly increased compared with the pre_high-intensity condition and post_control condition (Fig. 4, Extended Data Fig. 4-1).

**Table 5.** Repeated-measures ANOVA results for cortical excitability (TMS)

Parameters	Conditions	df	*F* value	*p* value
1 mV intensity	Condition	1	0.41	0.67
	Time	1	0.53	0.66
	Condition × time	1	1.2	0.3
AMT	Condition	1	2.68	0.08
	Time	1	2.8	0.07
	Condition × time	1	2.1	0.10
RMT	Condition	1	0.63	0.56
	Time	1	4.23	0.057
	Condition × time	1	0.44	0.3
I-O curve	Condition	1	3.92	0.067
	Intensity	3	3.24	0.07
	Time	1	1.73	0.18
	Condition × Intensity	3	4.67	0.061
	Intensity × time	3	1.26	0.13
	Condition × intensity × time	3	1.1	0.21
SICI-ICF	Condition	1	8.56	**<0.001**
	ISI	4	3.18	**0.001**
	Time	1	7.32	**<0.001**
	Condition × ISI	4	7.21	**<0.001**
	Condition × time	1	9.3	**<0.001**
	ISI × time	4	8.3	**<0.001**
	Condition × ISI × time	4	7.78	**<0.001**

Significant results at *p* < 0.05 are shown in bold.

**Table 6:** Mean and SD for TMS parameters before and after the different intervention conditions (low-, moderate-, and high-intensity exercise or control)

	Low intensity				Moderate intensity				High intensity				Control			
	Before	After	Change	*p*	Before	After	Change	*p*	Before	After	Change	*p*	Before	After	Change	*p*
TMS																
MEP (mV)	1.02 (0.2)	1.01 (0.5)	−0.0 (0.01)	0.21	1.02 (0.4)	1.01 (0.33)	−0.01 (0.02)	0.17	1.03 (0.23)	1.01 (0.35)	−0.02 (0.01)	0.16	1.01 (0.34)	1.04 (0.21)	0.03 (0.02)	0.23
AMT	37 (3.1)	38 (2.1)	1 (2.1)	0.32	39 (3.2)	38 (4.1)	−1 (1.1)	0.63	36 (3.1)	39 (2.1)	3 (1.2)	0.23	37 (4.1)	39 (7.1)	2 (1.2)	0.12
RMT	52 (5.1)	53 (6.8)	1 (1.2)	0.21	55 (6.12)	54 (7.1)	−1 (2.1)	0.432	55 (5.1)	59 (3.2)	4 (2.1)	0.75	56 (5.2)	58 (4.8)	2 (0.9)	0.54
SICI																
ISI 2 ms	−62 (23.1)	−63 (21.2)	−1 (2.3)	0.21	−65 (21.3)	−43 (18.2)	22 (21.1)	**0.038**	−65 (11.2)	−61 (10.2)	(1.3)	0.23	−62 (10.2)	−53 (11.2)	9 (2.20	0.74
ISI 3 ms	−59 (23.2)	−60 (20.2)	1 (2.2)	0.17	−60 (22.2)	−42 (19.3)	18 (17.2)	**<0.01**	−60 (21.0)	−58 (21.2)	(2.2)	0.21	−60 (21.2)	−59 (21.2)	1 (2.3)	0.66
ISI 5 ms	−81 (20.5)	−80 (17.2)	−1 (1.0)	0.16	−80 (19.8)	−56 (21.1)	24 (11.1)	**0.03**	−79 (11.3)	−88 (22.2)	−9 (3.1)	0.16	−70 (18.3)	−71 (21.3)	−1 (11.3)	0.87
General means of SICI	−67.1 (9.2)	−64.1 (9.2)	3 (2)	0.09	−66.2 (11.3)	−47.7 (13.4)	18.5 (2.2)	**0.04**	−66 (3.3)	−63.9 (8.2)	2.1 (1.3)	0.09	−67.3 (5.2)	−63.9 (7.3)	3.4 (1.1)	0.67
ICF																
ISI 10 ms	111 (24.5)	114 (21.2)	3 (1.9)	0.22	122 (25.2)	143 (20.1)	21 (21.2)	**<0.01**	121 (21.2)	134 (13.3)	22 (11.1)	**<0.01**	122 (11.2)	129 (13.4)	7 (12.2)	0.45
ISI 15 ms	130 (31.3)	133 (23.2)	3 (1.1)	0.21	132 (33.2)	195 (22.1)	63 (11.1)	**<0.01**	131 (13.2)	154 (11.2)	23 (21.2)	**<0.01**	131 (21.2)	132 (22.2)	1 (3.2)	0.82
General means of ICF	128.5 (11.2)	132 (9.1)	3.5 (2.1)	0.15	128.7 (10.2)	152 (11.3)	23.2 (12.2)	**0.04**	128.8 (12.1)	141 (13.2)	12.2 (3.1)	**0.04**	129 (20.1)	134 (12.2)	5 (2.3)	0.65

Significant results as pre-post comparison at *p* < 0.05 are shown in bold.

10.1523/ENEURO.0182-23.2023.f4-1 Figure 4-1 ***A***, Individual results preintervention and postintervention in each condition for SICI (as the mean percentage change from the unconditioned pulse) over ISIs of 2, 3, and 5 ms are shown. ***B***, Individual lines preintervention to postintervention in each condition for ICF are depicted (mean percentage change from the unconditioned pulse over ISIs of 10 and 15 ms). The asterisks indicate significant differences between preintervention and postintervention conditions. The hash symbols indicate significant differences between respective postconditions (*p* < 0.05). Download Figure 4-1, TIF file.

With respect to the association between aerobic exercise-induced effects on motor learning, and physiological parameters after interventions, there was only a significant negative correlation between SICI and motor learning RT (*r* = −0.73, *p* = 0.045) under moderate-intensity aerobic exercise. Reduced inhibition was thus associated with superior motor learning. No significant correlations emerged between motor learning RT and SI_1mV_ (*r* = −0.12, *p* = 0.92), RMT (*r* = 0.22, *p* = 0.42), AMT (*r* = 0.15, *p* = 0.98), I-O curve (*r* = −0.12, *p* = 0.92), and ICF (*r* = −0.35, *p* = 0.07) under moderate-intensity exercise. There were either no significant correlations between motor learning RT and SI_1mV_ (*r* = −0.11, *p* = 0.93), RMT (*r* = 0.21, *p* = 0.52), AMT (*r* = 0.14, *p* = 0.9), I-O curve (*r* = −0.11, *p* = 0.94), and SICI (*r* = −0.22, *p* = 0.67), ICF (*r* = −0.23, *p* = 0.45) under low-intensity exercise. Similarly, for the high-intensity aerobic exercise condition, motor learning RT and SI_1mV_ (*r* = −0.10, *p* = 0.92), RMT (*r* = 0.19, *p* = 0.82), AMT (*r* = 0.18, *p* = 0.81), I-O curve (*r* = −0.22, *p* = 0.77), SICI (*r* = −0.32, *p* = 0.071), and ICF (*r* = −0.21, *p* = 0.43) were not associated. No significant correlations between motor learning RT and SI_1mV_ (*r* = −0.1, *p* = 0.95), RMT (*r* = 0.18, *p* = 0.62), AMT (*r* = 0.15, *p* = 0.9), I-O curve (*r* = −0.12, *p* = 0.89), SICI (*r* =−0.12, *p* = 0.87), and ICF (*r* = −0.2, *p* = 0.72) emerged in the control condition.

## Discussion

In this study, we directly compared the acute effect of different aerobic exercise intensities on both motor learning and cortical excitability in sedentary individuals. The results indicate that aerobic exercise enhances motor learning and cortical excitability, and that the effect is nonlinear. Specifically, it was largest for moderate-intensity exercise, compared with low- and high-intensity exercise. Furthermore, a negative correlation between SICI and improvement of motor learning was shown selectively in the moderate aerobic exercise intensity condition.

In the SRTT, the motor learning performance did not differ between conditions before intervention. After intervention, the RT of the learned sequence was significantly shorter under the moderate-intensity exercise condition than under all other conditions. This result aligns with that of a foregoing study, in which a single session of moderate-intensity exercise improved learning of continuous tracking and motor sequence tasks (Snow et al., 2016). Moreover, in the present study, learning performance significantly differed between post_moderate-intensity aerobic exercise and the post_control condition, whereas no significant learning differences between the other two exercise conditions (post_low-intensity and post_high-intensity aerobic exercise) and the post_control condition emerged. Furthermore, the learning performance showed significant differences between the post_moderate-intensity and post_high-intensity/post_low-intensity aerobic exercise conditions. A previous meta-analysis reported similar results, indicating that moderate-intensity, but not low-intensity, aerobic exercise benefits motor learning (Northey et al., 2018). These study results thus extend previous findings that suggested nonlinear effects of exercise on motor learning.

With regard to corticospinal excitability, there was no significant differences in the pre_aerobic exercise condition. AMT, RMT, and the I-O curve did not show differences between conditions after intervention. This finding is in accordance with those of previous studies (Smith et al., 2014; Yamazaki et al., 2019). We found, however, enhanced ICF as well as a decreased SICI after moderate-intensity aerobic exercise. ICF is regulated predominantly by glutamate receptors with some GABA_A_ receptor contribution (Ziemann et al., 1995, 1996a). SICI is suggested to be primarily controlled by GABA_A_ receptors (Ilić et al., 2002; Paulus et al., 2008). These results indicate that moderate-intensity aerobic exercise exerts modulatory effects on excitatory glutamatergic neurotransmission and GABA-related inhibition. These findings are similar to those of animal studies showing that exercise decreases GABAergic inhibition and promotes glutamatergic facilitation (Yu et al., 2013; Arcangelo et al., 2017). Furthermore, previous studies in humans have shown that a single bout of moderate-intensity aerobic exercise increases ICF, whereas it decreases SICI (McDonnell et al., 2013; Mooney et al., 2016; Singh et al., 2014; Smith et al., 2014; Yamazaki et al., 2019). Our results align with these studies. In the present study, low-intensity aerobic exercise did not significantly change ICF and SICI, which is also in accordance with previous evidence (Singh and Staines, 2015; MacDonald et al., 2019). Moreover, here, high-intensity aerobic exercise enhanced ICF, whereas it did not change SICI. This result differs from those of a foregoing study that showed that high-intensity interval exercise decreased SICI, but had no effect on ICF (Stavrinos and Coxon, 2017). These heterogeneous results might be because our study chooses continuous high-intensity aerobic exercise in a sedentary population. Our findings show the novel observation of a nonlinear dosage-dependent effect of aerobic exercise on cortical excitability in sedentary individuals, which is similar in nonsedentary individuals (McDonnell et al., 2011; Singh et al., 2014).

Previous studies have demonstrated a nonlinear relationship between aerobic exercise intensity and motor learning performance (McMorris et al., 2003), which was shown also in the present study, and might be explained by the intervention dosage-dependent nonlinearity of physiological effects. In accordance with our results, a previous study revealed that the activation of the glutamatergic system was enhanced and GABAergic inhibition was reduced after moderate-intensity exercise (Kuo et al., 2023). Furthermore, a close association was found between the respective GABA reduction and motor learning improvement after moderate-intensity exercise (Kuo et al., 2023). In the present study, low-intensity aerobic exercise, which did not improve performance, also resulted in no physiological alterations, and thus might have been underdosed. The missing performance improvement observed in the high-intensity aerobic exercise condition, which was accompanied by reduced physiological alterations, compared with moderate-intensity exercise, might have been caused by cortical hyperactivity or counterregulation via GABA enhancement, as suggested by our physiological results. A major candidate mechanism for the impact of exercise on cortical excitability and cognitive performance might be GABA, not necessarily glutamate, since GABAergic inhibition was reduced, and glutamatergic facilitation enhanced under moderate aerobic exercise, which enhanced performance, but solely glutamate enhancement, as under high-intensity aerobic exercise, did not show this effect. Furthermore, aerobic exercise-induced decreases of SICI were associated with improvements in motor learning performance in the study, whereas there was no correlation between ICF and motor learning. This finding is similar with previous evidence, which showed that the magnitude of GABA reduction correlates positively with motor learning (Stagg et al., 2011). So far, studies have reported partially inconsistent results with regard to the effects of acute aerobic exercise on motor learning and cortical excitability (Audiffren et al., 2009; Gomez-Pinilla and Hillman, 2013; Singh et al., 2016; Snow et al., 2016; El-Sayes et al., 2020). The inconsistency of these results might be explained by differences of participant characteristics, which would not apply in our repeated-measures approach, exercise duration, the nature of the cognitive task, the time at which the task was administered in relation to exercise, and exercise modality. Given the proposed nonlinearity of effects, the limited consistency between studies is, however, not surprising.

### Limitations

This study has some limitations that should be taken into account. One limitation of the study is that we did not use a NeuroNavigator system to keep coil position constant, which might have resulted in an even higher precision of the coil position, and should be considered in future research. Since, however, motor thresholds and stimulation intensity required to induce an MEP size of 1 mV did not differ between sessions, we are confident that we had a reliable hot spot. Additionally, participants were allowed to use smartphones and read in the control condition, which might lead to confounding factors such as reading, Internet use, and others. Future studies should include a rest condition that mirrors the central aspects of the aerobic exercise protocol without the association with physiological exertion associated with deliberate exercise. Second, because motor learning performance was evaluated immediately after exercise, limited performance improvement might be detected because of a transient reduction in cerebral blood flow and fatigue, especially following high-intensity aerobic exercise (Lambourne and Tomporowski, 2010). Future studies should perform motor learning not only immediately after aerobic exercise, but also with some delay, or should assess performance repetitively over prolonged time courses. Furthermore, maintaining aerobic exercise intensity up to 95% of the HRmax over prolonged time courses is difficult. Thus, in future studies, researchers might want to consider adopting a high-intensity interval training protocol, which allows for high-intensity aerobic exercise interspersed with periods of active recovery. Finally, because the study was conducted in healthy sedentary individuals, its findings cannot be directly translated to clinical populations, in which transmitter availability and other features of brain functions might be different. Some studies have indicated that interindividual heterogeneity affects the relationship between aerobic exercise and cognitive outcomes. Studies should explore reasons for these heterogeneities and adapt intervention protocols, including individualization of interventions.

### Conclusions

This study indicates that aerobic exercise enhances motor learning and cortical excitability in sedentary individuals. Furthermore, these effects depended on the intervention dosage. Moderate-intensity, but not low- or high-intensity, aerobic exercise significantly enhanced motor learning and cortical excitability. The correlation hints for an association between SICI and motor learning, but is preliminary and needs further substantiation. The results of this study suggest that aerobic exercise is suited for cognitive/motor performance improvement, and that specifically moderate aerobic exercise might be best suited for performance improvement in sedentary individuals. To design and explore novel interventions for clinical applications, future studies should investigate how aerobic exercise intensity impacts more complex tasks and the effects of repetitive intervention, which might include personalization of respective protocols.

## Synthesis

Reviewing Editor: Frederike Beyer, Queen Mary University of London Decisions are customarily a result of the Reviewing Editor and the peer reviewers coming together and discussing their recommendations until a consensus is reached. When revisions are invited, a fact-based synthesis statement explaining their decision and outlining what is needed to prepare a revision will be listed below. The following reviewer(s) agreed to reveal their identity: Timothy Morris.

We have received comments from one reviewer, and as the second requested review has been too long overdue, have decided to process the manuscript with the single review received. Please see below comments. I agree with the reviewer that this is very interesting and potentially important work, but critical aspects of the methods need to be clarified. the reviewer also suggests some changes to how the results are presented, to make it easier for the reader to understand the data. Please see the detailed comments below.

Reviewer 1 comments:

Congrats to the authors for conducting a direct comparison of dose-response effects of different exercise intensities. This study was well needed however there are important details missing in the methodology and the results that limit the ability of the field to fully understand the protocols and reproduce the study. There are also some important methodological considerations that need to be addressed and/or answered for this study to contribute to the literature in this field.

Minor clarifications:

How was baseline heart rate determined (please provide methodology) and was this repeated every day before each intervention condition?

I am not familiar with the SRTT but based on the description given in the manuscript I am unsure how it is a “motor learning” task. Can the authors please provide more details or clarify how exactly participants responded to stimuli to make this a motor learning task and not simply a reaction time task? I see there is some more information about this in the stats section, please expand and move to the methods section. Consider a graphical depiction of the test.

Please can the authors justify the use of 80%aMT as the conditioning stimulus for SICI and IFC? Throughout the acute exercise - TMS literature there are some studies that use 80%aMT (e.g., Mooney et al., 2016) but a lot use 80% RMT. Per the Rossini et al., 2015 consensus guidelines on the use of TMS https://www.ncbi.nlm.nih.gov/pmc/articles/PMC6350257/

, the conditioning stimulus for SICI and ICF should be at an intensity that does not eveoke an MEP. The relation between the amount of inhibition (by SICI) and the conditioning stimulus is U-shaped whereby higher CS intensity less inhibition and eventual facilitation. Are the authors certain that 80%AMT did not elicit a supra-RM-threshold MEP?

Major clarifications:

How was the motor hotspot consistently maintained pre to post intervention? Can the authors be certain that the same cortical hotspot was stimulated pre and post intervention? Was any sort of neuronavigation used? It would be important to assure that the same cortical hotspot was targeted pre and post intervention and importantly, across intervention days.

Can the authors please justify the use of ADM as their muscle of choice? I know the literature on acute-exercise and TMS all use varying muscles to measure cortico-spinal excitability and reactivity, but I think the choice of muscle should be justified to help future studies better advance this area of research.

Please provide more details about the participant characteristics in Table 1.

Please report the results for all measures beyond the F value of the ANOVA in the results tables. It is important for the field to see mean and SD of the pre and post values and the change score for all variables (see Morris et al., Light aerobic exercise modulates executive function and cortical excitability, EJN, 2019 Table 1 and 2 as an example).

Please can the authors clarify if they checked and satisfied the statistical assumptions of analysis of variance? TMS data is often heavily skewed, which is not a violation of ANOVA, but can lead to non-normal residuals which would violate the assumptions of the ANOVA.

Can the authors please justify the use of student’s t tests and not controlling for multiple comparisons in the post hoc tests of significant interactions in the ANOVAs? Also, why is the term “exploratory” used when describing the post hoc test of ‘significant’ ANOVA effects in the statistical analysis section? It’s not exploratory if there is a significant main effect or interaction.

Major concern:

From figure 3 it is impossible to tell the direction or magnitude of change in either SICI or ICF as a function of exercise and these data are neither in any tables. As per my comment above, please transparently report all pre and post condition data for all measures and the change scores. In fact, based on figure 3 A and B, it sort of looks as if the condition/unconditioned MEP at short ISI (SICI) is higher after exercise (less inhibition), please can the authors clarify this and better depict the change in SICI and ICF as a function of the intervention in the figures. Consider representing the data as a % change from the unconditioned stimulus (i.e., unconditioned - CS/CS*100) to help readers grasp the direction and magnitude of the changes in these measures (this may require you to model the data separately across different ISIs- but I do not see how different ISIs add to the interpretation of the data). However, this is the most common way these data have been presented in the literature (see Mooney et al., 2016, Singh et al 2014, Morris et al., 2019) and for this paper to add to that literature the data should (and can) be presented in a similar way.

Please amend the figures to transparently show individual pre-post each condition data (See figures 3 and 4 in Morris et al., Light aerobic exercise modulates executive function and cortical excitability, EJN, 2019 as a good example). Representing the data as a percent change from unconditioned stimulus will help with this as the reader will be better able to see how each intervention changes the measure of cortical excitability/reactivity to each TMS measure, which is really the crux of the argument. The authors could consider representing the data both as individual lines pre to post intervention in each condition and as a % change between conditions in a bar plot so that readers can see the correct breakdown of each main effect and interaction of the statistical analyses.

Given the authors haven’t controlled for multiple comparisons of the simple bivariate correlations between task performance and TMS measures they should temper the conclusions paragraph regarding SICI being associated with motor learning as this likely wouldn’t pass a correction for multiple comparisons
