# Supplementary figures and images for: Acute Aerobic Exercise at Different Intensities Modulates Motor Learning Performance and Cortical Excitability in Sedentary Individuals
Source: eNeuro. 2023 Nov 16;10(11):ENEURO.0182-23.2023. doi: 10.1523/ENEURO.0182-23.2023 (PMC10668209; doi:10.1523/ENEURO.0182-23.2023)

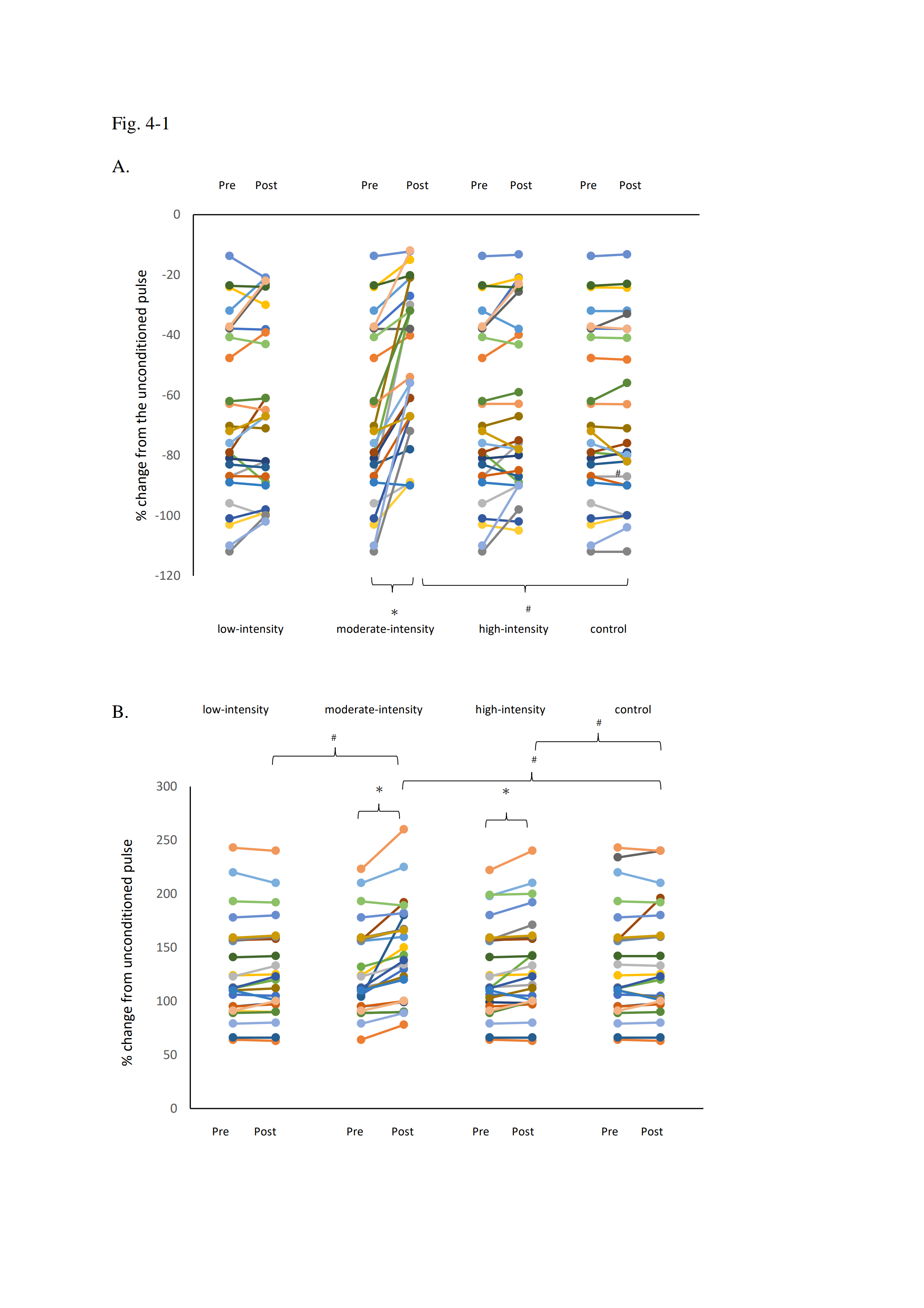

Supplement: Figure 4-1 — A, Individual results preintervention and postintervention in each condition for SICI (as the mean percentage change from the unconditioned pulse) over ISIs of 2, 3, and 5 ms are shown. B, Individual lines preintervention to postintervention in each condition for ICF are depicted (mean percentage change from the unconditioned pulse over ISIs of 10 and 15 ms). The asterisks indicate significant differences between preintervention and postintervention conditions. The hash symbols indicate significant differences between respective postconditions (p < 0.05). Download Figure 4-1, TIF file. [file enu-eN-NWR-0182-23-s01.tif]
